# Service staff encounters with dysfunctional customer behavior: Does supervisor support mitigate negative emotions?

**DOI:** 10.3389/fpsyg.2022.987428

**Published:** 2022-08-29

**Authors:** Biyan Xiao, Cuijing Liang, Yitong Liu, Xiaojing Zheng

**Affiliations:** School of Economics and Management, Weifang University of Science and Technology, Weifang, China

**Keywords:** dysfunctional customer behavior, customer misbehavior, negative emotion, prosocial service behavior, perceived supervisor support

## Abstract

Dysfunctional customer behavior is common in service settings. For frontline employees, negative encounters can cause short-term despondency or have profound, long-term psychological effects that often result in both direct and indirect costs to service firms. Existing research has explored the influence of dysfunctional customer behavior on employee emotions, but it has not fully investigated the psychological mechanism through which customer misbehavior transforms into employee responses. To maintain service quality and employee well-being, it is important to understand the impact of customer misconduct on employee emotions and its effect on subsequent service behavior. To assess the process through which dysfunctional customer behavior manifests as negative emotions in frontline service employees, and the influence of negative employee emotions on their prosocial service behavior, we surveyed 185 frontline banking service employees. We sought information on service employee experiences, attitudes, and feelings regarding dysfunctional customer behaviors, the perceived level of supervisor support, and employee prosocial service behavior intentions. Structural equation modeling and hierarchical linear modeling were used for statistical analysis and hypothesis verification. Results indicate that dysfunctional customer behavior has a positive relationship with bank service employee negative emotions and a negative influence on employee prosocial service behavior. The study found that negative emotions fully mediated the relationship between dysfunctional customer behavior and prosocial service behavior. The moderating role that perceived supervisor support plays on the relationships between dysfunctional customer behavior and negative emotion was also investigated. The results show that perceived supervisor support moderates the relationship between dysfunctional customer behavior and negative employee emotions. Finally, the study provides bank managers with effective strategies to assist frontline employees to manage and deter dysfunctional customer behavior, and presents employees with internal recovery strategies when encountering dysfunctional customer behavior.

## Introduction

From a marketing perspective, the exchange relationship between marketers and consumers is often considered to be positive and mutually beneficial (Cadeaux, 2000), as consumers’ needs are met by consuming products or services that marketers promote, who, in turn, derive desired economic benefits from consumer consumption (Vargo and Lusch, 2004). However, consumers are not always well-intentioned in the exchange relationship (Wilson et al., 2022), often portraying a dark, negative side, such as shoplifting (Cox et al., 1990; Egan and Taylor, 2010), queue jumping (Adam, 2021), vandalizing (Fisk et al., 2010), gratuitous complaints (Reynolds and Harris, 2005) and spurious product returns, verbal and physical abuse of employees and fellow customers (Harris and Daunt, 2013), and abuse of company resources (Schaefers et al., 2016). Individuals who exhibit such behavior are called “jaycustomers” (Harris and Reynolds, 2004), problem customers (Bitner et al., 1994), and dysfunctional customers (Gong et al., 2014), and the behavior they display referred to as deviant customer behavior (Daunt and Harris, 2011), dysfunctional customer behavior (Harris and Reynolds, 2003), or customer misbehavior (Fullerton and Punj, 2004; Gursoy et al., 2017). These kinds of dysfunctional customer behaviors can cause material losses and psychological damage to product manufacturers, marketers, other consumers (Fullerton and Punj, 2004), and employees. Studies to date concerning dysfunctional customer behavior mainly address the antecedents of the behavior (McColl-Kennedy et al., 2009), its diverse forms (Fullerton and Punj, 2004; Harris and Reynolds, 2004; Berry and Seiders, 2008; Funches et al., 2009; Reynolds and Harris, 2009), the motives or drivers of the behavior (Reynolds and Harris, 2005; Daunt and Harris, 2012), its consequences (Harris and Reynolds, 2003) and strategies to manage it (Dootson et al., 2018).

Although researchers and practitioners agree that dysfunctional customer behavior is a universal phenomenon in service situations (Gong et al., 2014; Schaefers et al., 2016; Boukis et al., 2020), it occurs frequently and has serious consequences for service firms, other customers, and employees. Service firms seeking customer satisfaction as their strategy for competitive advantage require frontline service employees to exhibit superior qualities and capabilities when providing face-to-face services, such as impression management and considerate behavior. Such firms are likely to expect that employees display the behaviors desired by the organization and the customer, rather than acknowledge the emotions frontline service employees actually experience, as firms work hard to continually recruit employees able to perform the expected behaviors. Further, service firms are predominantly focused on identifying and addressing the factors that cause customer dissatisfaction, exhibiting indifference to the emotions that employees may feel in their service encounters. With regard to service-oriented employees, enduring rude customer behavior with compromise and a forgiving manner has become part of their jobs, and to fulfill the primary goal of satisfying customer needs and providing quality customer service, customer misconduct is likely to be ignored or even forgiven and accepted (Fellesson and Salomonson, 2020). Managers generally do not regard customer misbehavior as having an adverse impact on employee physical and mental health and behavior. Moreover, managers may try to control or restrain employees’ emotional responses in service encounters using standardization and strengthening of norms to force employees to provide a level of customer service that the firm and customer deem satisfactory.

In many service settings, dysfunctional consumer behavior either directly or indirectly influences unsatisfactory service encounters (Huang et al., 2010). Through the influence of service mantras, such as “The customer is always right” or “The customer is king,” proclaimed in the era of customer sovereignty, customers often have a sense of superiority when encountering service personnel and lack respect and understanding for service staff. Within the service interaction, once problems and disputes arise, the “customer first” service principle can place enterprises and service personnel in a weakened position. Additionally, to achieve profit goals, many service firms attempt to manage or regulate their employees’ emotions. Where firms blindly insist that all employees should be kind, smiling, and humble, no problems arise if the customer is friendly; if staff encounter unfriendly customers, and the situation requires them to maintain an ingratiating demeanor, such work requirements become a form of work pressure for frontline employees.

Job-related stress can lead to negative physiological, psychological, and behavioral responses among employees. Growing evidence suggests that frontline service employees exposed to dysfunctional customer behavior will try to alleviate the impact of the negative emotions they experience (Muraven and Baumeister, 2000). They may fall into an adverse mental state through constant contemplation about the incident, finding it difficult to regulate their behavior in the absence of opportunities to take revenge on the customer (Wang et al., 2022). Customer incivility causes employees to become angry (Domagalski and Steelman, 2005), which can affect normal work, and such incivility can trigger negative emotions causing the employee to retaliate against the customer (Walker et al., 2014). These negative emotions can influence employee attitudes and may cause problems in the way they are handled. For instance, negative emotions can spread within the enterprise like an invisible virus, affecting the atmosphere of the entire firm when the problem is serious. Further, if employees’ negative emotions are not properly dealt with and released, poor service performance or even retaliation against customers and the organization can occur that can result in a negative impact on turnover and may even affect the sustainable management of the organization. In this way, customer misbehavior can increase direct and indirect costs to the company, resulting in a decline in corporate performance (Harris and Reynolds, 2003). Therefore, to satisfactorily address this dynamic, enterprises must view customer relations as part of the production process, as a production resource, and, similarly for employees, as a human resource.

To enhance customer satisfaction at the point of service encounter, managers of service firms have endeavored to control and prevent the dysfunctional behavior of employees at customer contact, while giving limited attention to the negative consequences of customer misbehavior (Harris and Reynolds, 2004). In recent years, with an increasing proportion of economic development coming from the service industry, the status of employee emotions is becoming a central element in service quality management (Slåtten, 2008). Job-related emotions in the service workplace, in particular, are becoming a much-discussed topic, drawing the focus of both managers and practitioners. Academic research is turning its attention to the profound negative impacts of customer dysfunctional behaviors on service employees’ emotions.

Evidence suggests that an individual emotional reaction is usually not the end of the story. Service employee reactions to dysfunctional customer behaviors may influence subsequent service behavior (Amarnani et al., 2019). If employees experience anger (Rupp et al., 2008; Spencer and Rupp, 2009), anxiety (Wang et al., 2013), restlessness, depression, frustration, burnout, or other negative emotions (Harris and Daunt, 2013; Walker et al., 2014), and if these negative emotions are not alleviated, they could lead to employee physical or mental health disorders (Baranik et al., 2017), emotional disorders (Madupalli and Poddar, 2014), and emotional exhaustion (Baeriswyl et al., 2016; Alola et al., 2019) at work. These reactions, triggered by negative emotions, can have direct and significant effects on perceived customer service quality and business performance. Finding ways to help frontline service employees to effectively deal with dysfunctional customer behavior, therefore, is a key issue for both researchers and practitioners. Some scholars have found that the support of leaders can help to enhance the emotional bonds of employees to the organization, reduce their work stress, and increase positive emotional attitudes and behaviors (Tian et al., 2014). A supportive work environment plays an important role in reducing workplace stressors and improving job performance.

Importantly, support from leaders or supervisors can disrupt the psychological link between dysfunctional customer behavior and negative employee emotion by attenuating or preventing stress assessment responses. One strand of research focuses on helping employees to mitigate negative emotions or restore their equilibrium subsequent to a customer misconduct encounter. However, how frontline service employees actually behave once their negative emotions have been triggered by dysfunctional customer behavior is still a neglected area of research. Little research has also been undertaken on ways to regulate or mitigate the mental and physical health impact on employees as a result of customer misbehavior, and many strategies remain to be explored in depth. This current study aims to extend this broad research topic by examining the impact of negative emotions on frontline employee service behaviors triggered by dysfunctional customer behavior. In addition, the research will explore whether perceived supervisor support moderates the impact of dysfunctional customer behavior on frontline service employee negative emotions, which, in turn, is predictive of service behavior.

## Literature review

### Dysfunctional customer behavior

Customer behaviors that violate generally accepted social and service exchange norms are commonly seen in daily service encounters. Customers who exhibit irrational or illegal behavior have been called jaycustomers (Harris and Reynolds, 2004), problem customers (Bitner, 1992; Madupalli and Poddar, 2014), and wrong customers (Woo and Fock, 2004). Dysfunctional customer behaviors have been described variously as deviant consumer behavior (Reynolds and Harris, 2006; Wilson et al., 2022), aberrant consumer behavior (Amasiatu and Shah, 2014), dysfunctional customer behavior (Harris and Reynolds, 2003), consumer misbehavior (Fullerton and Punj, 2004), unethical consumer behavior (Babakus et al., 2004), and disruptive customer behavior (Cai et al., 2018). Based on a composite of these different terms and related definitions, this study defines dysfunctional customer behavior (DCB) as that which intentionally or unintentionally causes trouble, inconvenience, or problems to service firms, employees, or other customers.

Currently, a number of comprehensive conceptual frameworks are devoted to understanding the triggers and motivations of customer misconduct and the underlying psychological processes involved. Importantly, they reveal the contextual factors surrounding such customer misbehavior, the different types and consequences, and the means to deter it (Harris and Reynolds, 2003; Fullerton and Punj, 2004; Daunt and Harris, 2011, 2012; Anaza and Zhao, 2013; Dootson et al., 2018; Tan et al., 2020). Dysfunctional customer behavior can be directed toward employees (e.g., through verbal or physical abuse, sexual harassment, or violence), and these behaviors can have negative impacts on employees both cognitively and behaviorally. For instance, they can cause negative emotional outcomes, such as anger, anxiety, and depression (Yagil, 2008; Harris and Daunt, 2013) and emotional exhaustion and emotional dissonance (Sliter et al., 2010; Madupalli and Poddar, 2014), damage employees’ job performance (Baranik et al., 2017), increase turnover intentions (Poddar and Madupalli, 2012), and result in job burnout (Han et al., 2016). Such dysfunctional behaviors not only spoil other customers’ consumption experience (Harris and Reynolds, 2003) and decrease other customers’ satisfaction (Grove and Fisk, 1997), perhaps what is worse, they can be contagious and cause other customers to imitate such dysfunctional behaviors, for example, by cutting the queue, performing hostile physical acts, and complaining vociferously (Harris and Reynolds, 2003; Fullerton and Punj, 2004). For firms, dysfunctional customer behavior, such as shoplifting, fraudulent returns, copyright infringement, fraud, vandalism, etc., all indirectly or directly contribute to the organization’s financial costs (Harris and Reynolds, 2003). Moreover, Gong and Wang (2021) argue that dysfunctional customer behavior can have a negative impact on brands, such as brand-negative word-of-mouth (WOM), brand boycotting, and brand retaliation.

### Negative emotion

Emotion, affect, feeling, etc., are widely used terms in psychology and in consumer behavior. These concepts can often use interchangeably in studies (Sundie et al., 2009), although Holbrook and Batra (1987) argue that “affect” refers to pleasant or unpleasant sensations experienced under the stimuli and circumstances of everyday life that are closely related to cognitive processes. “Emotion,” on the other hand, refers to a series of physiological behavioral responses to external stimuli and differs from affect in that emotions are not simply pleasant or unpleasant, but complex and comprehensive experiences.

Based on the difference in valence, studies generally classify emotions as positive or negative (Watson et al., 1988; Van Dolen et al., 2001; Chebat and Slusarczyk, 2005). In this study, we focus on negative emotions that are triggered by either external or internal factors of specific behaviors that are often accompanied by anger, anxiety, disgust, sadness, fear, frustration, harm, guilt, shame, and disappointment. These negative emotions can be detrimental to an individual’s normal thinking processes and the smooth completion of a task or job (Yu et al., 2021). The deeper the negative emotion, the more people tend to exhibit worry and sensitivity, and unpleasant emotional states, such as anger, hatred, and uneasiness, will often occur. If a person experiences a negative emotion, they tend to exaggerate the extent of the problem more than it is actually perceived (Van den Bos, 2003). If such negative emotions are not discharged and released in a timely and appropriate manner, negative effects will be generated (Watson et al., 1988).

In a service industry, characterized by frequent interpersonal interactions, frontline employees encounter many customers daily and perform emotional labor. For people who engage in this work of constantly talking to strangers and satisfying their requirements, the person’s psychological and physical fatigue increases, and there is a tendency to display psychological responses, such as feelings of powerlessness, nervousness, and social phobia, that, in turn, are manifested as indifference or hostility toward customers (Chi et al., 2013). Customers’ negative tones, words, actions, and unreasonable demands on frontline employees increase the chances of experiencing negative emotions and can cause affected employees to retaliate to repair their self-esteem (Sommovigo et al., 2020).

### Prosocial service behavior

Bettencourt and Brown (1997) first proposed the concept of prosocial service behaviors (PSBs), defined as service employees’ helping behavior directed at either customers or coworkers. These behaviors are usually spontaneous, extend beyond formal role requirements, and are positively associated with customer relationship management and organizational performance. Specifically, frontline service employee PSBs have a significant impact on the perception and satisfaction of customers in terms of service quality (Bettencourt et al., 2005). They also influence a customer’s loyalty or decision to change service providers, which, in turn, affects organizational efficiency and performance (Podsakoff et al., 2009). The concept of PSBs is now well established and has a high profile in service marketing and management research.

Based on the research of Bettencourt and Brown (1997), PSBs can be divided into three domains, namely: role-prescribed customer service behavior, extra-role customer service behavior, and cooperation behavior. Firstly, role-prescribed customer service behavior refers to expected service behaviors when a frontline service employee serves customers (Tsaur et al., 2014). A customer’s expectation of employee service behavior may come from implicit norms and requirements in the service workplace, or from explicit obligations set out in documents that the organization develops, such as job descriptions and performance evaluation forms (Brief and Motowidlo, 1986). Expectations may include delivering accurate information about the product or service, being polite and smiling, and showing gratitude and appreciation to customers, etc. These service behaviors will have a positive effect on customer perceptions of service quality, satisfaction, loyalty, and sales performance (Bitner, 1990). The second domain, extra-role service behavior, refers to the discretionary behaviors that a service employee provides to a customer that exceeds the requirements of the formal role (Bettencourt and Brown, 1997). These discretionary actions may impress and delight customers by providing them with extra attention and concern and by spontaneously providing exceptional customer service. The last domain, cooperation behaviors, refers to the helpful behavior of service employees in assisting colleagues to complete their service in the workgroup (Bettencourt and Brown, 1997). Cooperation behaviors emphasize a willingness to help other employees of the organization, such as voluntarily helping those who are not adapted to the work environment or who are having difficulties in the workplace, which, in turn, enhances organizational teamwork and creates high value for external customers.

### Perceived supervisor support

Support within the workplace affects not only organizational performance, but also individual performance and development. In particular, supervisor support plays an important role in regulating the stress and emotions of organization members (Beehr and Gupta, 1978). Perceived supervisor support (PSS) refers to the degree to which employees perceive their supervisors as caring about their well-being and the extent to which supervisors value their organizational contribution (Maertz et al., 2007). This means that, in the process of the employee completing their work and performing their functions, the supervisor should recognize subordinates’ achievements, develop their career plans, and help them to achieve their career goals (Greenhaus et al., 1990; Eisenberger et al., 2002).

Supervisors who have empathy and respond appropriately to employees’ needs are particularly successful at managing their employees’ emotional responses (Cole et al., 2006). To encourage members to act voluntarily and proactively, supervisors should give value and meaning to the contributions of employees, provide timely assistance to employees when they encounter difficulties, and provide them with the resources they need to perform their tasks, including giving regular feedback (Oguegbe et al., 2017). Thus, supervisors can play a key role in improving employee competence and promoting their career development (Cromwell and Kolb, 2004; Hezlett and Gibson, 2005). Further, perceived supervisor support can affect employee perceptions and attitudes toward the organization as a whole (Rhoades and Eisenberger, 2002). Receiving this support from superiors allows organizational members to immerse themselves in and engage with their current organization and work. In contrast, if employees feel that they are not receiving enough support, this can lead to a decline in their confidence about their abilities and their potential (Higgins, 2000).

Scholars vary with regard to the specific behaviors or constituent factors that supportive supervisors exhibit, although supervisor support is generally considered to consist of four factors: emotional support, assessment support, instrumental support, and informational support (Cohen and Wills, 1985; Parker et al., 2003). Emotional support is the care and concern a supervisor gives in the execution of work or professional life, including support to overcome mental difficulties and is mainly formed through personal networks that encompass trust, respect, intimacy, concern, listening, and enhancing well-being (Zeng et al., 2021). Assessment support is the supervisor’s encouragement and recognition of work, including identifying difficulties, providing work feedback, respecting different personalities, recognizing talent, respecting values and praising, etc. Instrumental support refers to providing funds or goods needed to perform a job and so helping employees to improve performance. Informational support refers to providing needed information or advice to solve problems or to help individuals solve problems and improve work performance. Because frontline employees experience frustration and stress when dealing with dysfunctional customers, the emotional and appraisal support of supervisors is a necessary element of service work.

## Research framework and hypotheses

### Conceptual model

The research framework was developed to assess negative emotions triggered by dysfunctional customer behaviors that affect frontline employees’ service behaviors. The model is based on the mediation effect of negative emotions, and the moderation effect of perceived supervisor support. The model proposed to investigate the hypotheses developed is illustrated in Supplementary Figure 1.

### Research hypotheses

#### Dysfunctional customer behavior and employees’ negative emotions

Employees assess specific situations through personal cognition and produce corresponding emotional responses, so that dysfunctional customers’ negative words, deeds, and unreasonable demands on employees increase the likelihood that they will experience negative emotions (Spector and Fox, 2002). In the process of service encounters, frontline employees and customers experience empathy as part of the interaction, so that customers’ words and deeds, attitudes, emotions, etc., can easily affect the emotions of employees. Customer misconduct, such as verbal abuse, can make employees feel depersonalized, and create feelings of negativity and being disrespected (Dormann and Zapf, 2004). Further, customers can deliberately interrupt the provision of services, engage in misconduct against service providers, and, in severe cases, even abuse, beat, and exhibit other non-social behaviors (Reynolds and Harris, 2006). Even though such situations sometimes occur in service scenarios, employees are required to maintain comfortable and positive interactions with customers, which can lead to employees bearing excessive emotional burdens and experiencing serious consequences, such as mood disorders, emotional exhaustion, absenteeism, and resignation as a result of negative encounters.

Based on a questionnaire survey of call-enter employees, Wang et al. (2013) found that if a service employee received more customer mistreatment on particular days, he or she ruminated more on the negative customer encounter in the evening, which, in turn, led to higher levels of negative emotions experienced the next morning. It follows that customer mistreatment of employees can damage their short- and long-term emotional well-being (Harris and Reynolds, 2003). Therefore, if frontline employees suffer from customers’ unfriendly tones, words, deeds, and unreasonable demands or actions, the likelihood of negative emotions increases. At the same time, the misbehavior of customers will result in frontline employees experiencing psychological pressure, such as being humiliated from rude, aggressive, and threatening customer behavior, which can have a negative impact on the service employees’ emotional reactions.

Tan et al. (2020) indicate that daily jaycustomer behavior triggered anger and anxiety, if a service employee encountered aggressive and uncivilized customers. Such experiences can have a negative impact on emotional responses, leading to an increase in emotional exhaustion. Thus, when frontline service employees perceived they were being treated uncivilly or rudely, they may experience immediate negative emotions, such as irritation, anger, indignation, and feeling disrespected within service workplaces (Lazarus, 2020). Sliter et al. (2012) also argued that dysfunctional customer behaviors develop negative emotions among staff that affects their work behaviors. McCance et al. (2013) found a direct influence of dysfunctional customer behavior on the emotional responses of employees. Based on this discussion, we hypothesize that:

*H1*: Dysfunctional customer behaviors play a positive role on frontline service employees’ negative emotion.

#### Dysfunctional customer behavior and prosocial service behavior

In general, frontline service employees will try to understand the reasons for a customer’s dissatisfaction and help them to solve problems, except if the behaviors are unusually negative and undesirable, offensive, aggressive, frightening, personally insulting, or abusive (Mattar, 2021). Then, employees will feel psychologically and physically exhausted, and, if supervisors blindly emphasize the meeting of required professional standards, employee work motivation will be lost, work pressures will increase, and feelings of disgust and resistance will rise. According to the frustration-aggression theory, unfair events experienced in the workplace can trigger frontline service employee deviant behavior, which, in turn, will manifest in aggressive behavior (Fox and Spector, 1999). Dysfunctional customer behaviors experienced by frontline service employees can be considered as unfair events in the workplace (Peng et al., 2021). From this perspective, we argue that frontline service employees who encounter customer misbehavior may behave in a manner inconsistent with their role requirements. Meanwhile, because of professional requirements, frontline service employees can choose only to tolerate or remain silent, even if they encounter customer misconduct, as customers are the very people whom they are eager to assist and serve (Grandey et al., 2004). These requirements can increase employee emotional dissonance and exhaustion, which will have a negative impact on their willingness for prosocial service behavior.

Frontline service employees are often faced with a variety of unpredictable situations, which correspond to stressors, from which they are prone to experience emotional disorders or exhaustion in the workplace. This can adversely affect employee physical and mental health (Hwang et al., 2021). Therefore, dysfunctional customer behaviors are one of the key predictors driving frontline service employee sabotage action (Yagil, 2021), such as joking about customers or colleagues to please themselves, neglecting to comply with company rules and regulations, adjusting the speed of service according to one’s own emotions or personal needs, expressing hostility, anger, or frustration to customers, deliberately delaying service depending on employees’ moods and emotions, deliberately making inappropriate remarks or responses to customers, retaliating against customers in a rude manner, etc. (Harris and Ogbonna, 2009; Chi et al., 2013; Lee and Ok, 2014). From this perspective, most theorists hypothesize that, when customers treat frontline service employees unfairly, they might retaliate on customers by disrupting the service encounter or jeopardizing the service quality (Harris and Ogbonna, 2009; Van Jaarsveld et al., 2010). Based on this discussion, we hypothesize that:

*H2*: Dysfunctional customer behaviors play a negative role in frontline service employees’ prosocial service behavior.

#### Negative emotion and prosocial service behavior

According to affective events theory, those events experienced by service employees in the workplace influence their personal emotional responses, which, in turn, affect their service behavior (Chen et al., 2022). Emotions are a series of responses to stimuli in the external environment that induce changes in people’s behavior. If people have positive emotions, they often have a positive attitude when recalling events, and analyze actions or situations positively. In view of this, in the service industry, where interactions occur frequently, emotions can be a trigger for change in employee service behaviors. Employees in the service industry engaged in emotional labor can experience both positive and negative emotions caused by customers misbehavior in the process of their work. Employees who experience negative emotions are more likely to show indifference to customers and to be hostile compared with employees who do not have such experiences (Morris and Feldman, 1996). The negative emotional state of frontline service employees will affect their work status and attitudes, which, in turn, will affect their role behavior (Yao et al., 2019). If frontline service personnel are in a negative mood, they are more likely to maximize short-term results by cheating customers or resorting to sales techniques and appearing indifferent to customers’ needs. For example, customer-induced frustration causes employees to generate counterproductive behavioral responses (Fox and Spector, 1999) that increase their job burnout (Keenan and Newton, 1984), lower the quality of their service performance (Child and Waterhouse, 1953), and reduce customer perceived-service quality (Slåtten, 2010).

A frontline service employee’s willingness to get close to customers means that they will try to understand and meet the needs of the customer effectively. Employees who are in a good position to deliver what is expected and desired by the customer increase the chance of satisfying the customer. Negative emotions induced by dysfunctional customers, conversely, could hinder a frontline service employee’s willingness and ability to get close to the customer. Frontline service employees who experience dysfunctional customer behavior may take retaliatory action, such as delaying service delivery processes openly or covertly (Harris and Ogbonna, 2009). Through group interviewing, Szczygiel and Bazińska (2021) found that when extended behavioral coping occurred, regardless of whether the situation was resolved, service employees were less willing to interact with subsequent customers. This was especially the case when frontline service employees felt that a customer treated them unfairly, in which case they were likely to express hostility during the service delivery encounter (Groth and Grandey, 2012) and were unwilling to assist their coworkers in providing customer service. Thus, we hypothesize that:

*H3*: Negative emotions triggered by dysfunctional customer behaviors have a negative impact on employees' prosocial service behavior.

#### The mediating role of negative emotion

Scholars believe that emotions have a certain predictive effect on individuals’ behavior, especially negative emotions (Yu et al., 2021). The negative emotion frontline employees experience can affect their subsequent service attitude and thoughts, which then affect their in-role and extra-role service behaviors (Wirtz and Jerger, 2016). Thus, this study takes the view that negative emotions triggered by dysfunctional customer behaviors will have a negative impact on service behaviors. According to the affective events theory, specific work events trigger employees’ emotional reactions that further affect their attitudes and behaviors (Chen et al., 2022). Therefore, emotions can be seen as a bridge between the characteristics of the service environment and employees’ service behavior. Therefore, the specific event that employees experience in the workplace will firstly stimulate emotional reactions, and these emotions will then affect their subsequent behaviors.

As Borman et al. (2001) demonstrate, emotions, such as worry, anxiety, and anger, have a significant mediating effect on stressors, counterproductive behavior, and organizational citizenship behavior. Yu et al.’s (2021) empirical research conducted *via* survey of financial company employees found that negative emotion mediated the relationship between employees’ job insecurity and extra-role behavior. Other researchers have suggested that, with corresponding negative emotion, significant negative correlations exist between dysfunctional customer behaviors and employees’ service behaviors. For instance, when employees encounter customer misbehavior in the workplace, their role requirements and their inner emotions conflict, which causes frustration, sadness, and even fear, and employees will feel that it is difficult to again provide services with a smile. They then begin to snub customers’ requirements and turn a blind eye to customer or coworker needs for help. Further, they will become less friendly and more aggressive in the workplace, which, in turn, adversely affects their interpersonal relationships and work communication. These research results maintain that a negative emotion can affect service employee behavior. Therefore, we infer that negative emotion plays a mediating role on the relationship between dysfunctional customer behaviors and employee service behaviors. Based on this discussion, we hypothesize that:

*H4*: Negative emotion has a mediating effect on the relationship between dysfunctional customer behaviors and prosocial service behaviors.

#### The moderating effect of perceived supervisory support

As indicated, support in the workplace affects not only organizational performance, but also employee’s individual performance and development. Perceived supervisor support plays a significant role in regulating stress and emotions among members (Beehr and McGrath, 1992). To stimulate the spontaneity and initiative of employees, organization can value their contribution, help them in times of difficulty, provide resources to complete work tasks *via* their direct supervisor, and receive regular feedback. In an uncertain work environment, employees need to be aware that their input is being considered, that frequent and accurate feedback is being given, and that resources are available when they need them. Therefore, we infer that supervisors’ support plays a vital role in moderating employees’ experiences of customer misbehavior.

Research findings suggest that managers who are empathic and responsive to their employees’ needs are more effective in moderating their subordinates’ emotional reactions (Humphrey, 2002; Cole et al., 2006). Shanock and Eisenberger (2006) highlight the importance of organizational and supervisor support, emphasizing that, if members recognize that their immediate supervisors understand their work and the position they are in, and provide them with material aid and emotional support, counter productive work behavior toward the organization can be reduced. Supervisors can also help employees reduce work-related stress through programs that offer emotional support (Han et al., 2016). Halbesleben’s (2006) meta-analysis indicated that supervisor support plays a significant and important moderating role in reducing various job stressors in the workplace, arguing that employees with high perceived organizational support were more likely to experience positive emotions than those with low perceived organizational support. Han et al. (2016) found that organizational and supervisory support plays a significant role in alleviating the connection between uncivil customer behavior and employee burnout. Thus, based on these research findings, we infer that perceived supervisor support plays a significant moderating role when frontline service employees encounter dysfunctional customer behaviors, specifically that perceived supervisor support will significantly decrease service employees’ negative emotion stemming from dysfunctional customer behavior. We therefore hypothesize that:

*H5*: Perceived supervisory support will moderate the relationship between dysfunctional customer behaviors and frontline service employees’ negative emotion.

## Methodology

### Measurement items

The main measurement variables used to test the research model and hypotheses include dysfunctional customer behavior, negative emotion, and prosocial service behavior of bank tellers, and perceived supervisor support. The scales for these four variables are all measurement items that are commonly adopted and used in existing studies. Since the variables used in this study are abstract concepts, we have defined them in an operational manner. In addition, validated measurement items in relevant studies have been modified and supplemented according to the purpose and theme of this study.

Based on the definition of Harris and Reynolds (2003), in this study, the operational definition of dysfunctional customer behavior is the customer behavior that intentionally or unintentionally disrupts service processes and negatively affects the organization, the service employees, and other customers. The measurement items of dysfunctional customer behavior are based mainly on the eight types of poor customer behavior proposed in the research of Harris and Reynolds (2003). We removed the items that occur less frequently in the bank workplace, and, finally, according to the needs of this study, a total of 15 items were measured from the three aspects including: insulting words, rude behavior, and rude attitudes. The operational definition of negative emotions is as follows: when frontline service employees encounter inappropriate customer behavior, they experience emotions, such as anger, anxiety, disgust, sadness, fear, frustration, hurt, guilt, shame, and disappointment. Negative emotions in employees were measured using 15 items only about negative emotions from the Job-related Affective Well-Being scale presented in the study of Van Katwyk et al. (2000). For prosocial service behavior, we define it as voluntary service behavior of bank tellers, which aims to provide better service for bank customers and improve organizational performance by helping customers and colleagues. Employee prosocial service behavior was revised and improved according to the 15 items that Bettencourt and Brown (1997) proposed and have been used as measurements in this study. The operational definition of perceived supervisor support is the understanding, concern, recognition, and support of superiors given to the frontline service employees when they encounter customer misbehavior. The items used for measuring perceived supervisor support are those developed by Paille et al. (2013). The variables measured in this study were determined using a Likert five-point scale. To verify the hypotheses, SPSS v.24.0 and AMOS v.24.0 software were used for data analysis.

### Data collection and participant demographics

The questionnaire data of this study were mainly collected from bank tellers of the Qingdao Agricultural Bank and the Industrial and Commercial Bank of China. As bank tellers work directly with customers every day, employees in this type of job are at risk for experiencing dysfunctional customer behavior. Before administering the questionnaire, we conducted a pre-survey test and found that the Cronbach α value for all variables was above 0.80. The questionnaire was distributed *via* a direct visit, with five to eight copies being given to each bank branch. A total of 200 copies were randomly distributed in this way. Of these, a total of 187 questionnaires were returned, two of which were invalid, leaving 185 questionnaires to be used for statistical analysis. Specifically, the composition of the sample is 42.70% male, 57.30% female; 24.90% of participants had an age range between 21 and 30 years, 35.10% between 31 and 40 years, 31.40% between 41 and 50 years, and 8.6% over 50 years. In terms of academic qualifications, the proportion of college graduates is the highest with 69.70%, and 33.50% of respondents had worked for more than 5 years, which constituted the largest proportion of respondents.

## Results

### Common method variance test

According to Podsakoff et al. (2003), Common method variance (CMV) is a potential measurement error that has a serious adverse effect on the validity of the results of a study. The data collected in this study were in the form of a questionnaire, self-completed by respondents, within which respondents may have unconsciously tried to maintain consistency in their answers or provide the ideal answer, potentially leading to statistical measurement errors and biased results, or distortion of the true relationship between concepts (Podsakoff and Organ, 1986). The method used to measure the presence of CMV is Harman’s (1976) one-factor test. According to this method, exploratory factor analysis is performed, but unrotated factor analysis is used. If there is a significant common method variance, two conditions must be met. Firstly, only one factor is extracted from the factor analysis, or, secondly, one factor accounts for a significant proportion of the total variance (Podsakoff et al., 2012).

In this study, an exploratory factor analysis was performed on variables, such as dysfunctional customer behaviors, negative emotions, prosocial service behavior, and perceived supervisor support, and the unrotated factor analysis and varimax rotation method were applied to principal component analysis. The results extracted nine factors, all of which had eigenvalues above 1.0. These nine factors account for 72.472% of the total variance, with the first factor (31.206%) not accounting for the major part of the total dispersion. Therefore, no errors existed, according to common method variance (CMV).

### Reliability and validity analysis

To verify the reliability of the measuring items, the study uses the Cronbach’s α value. Supplementary Table 1 displays the reliability of variables, the means, and standard deviations of this study. The Cronbach’s α, the most commonly used method of internal consistency analysis, indicates in the table below that all values for the variables in this study are above 0.7, and, thus, all variables measured in our study have good reliability.

### Confirmatory factor analysis

#### Convergent validity

To verify the reliability of the measured variables in this study, the CFA (confirmatory factor analysis) test was performed. The CFA is used mainly to verify the factor structure of the observed variable, and to confirm if the observed variable can measure the latent variable precisely. This study uses convergent validity and discriminant validity analysis to examine the goodness of fit index. Although there are slight differences of opinion between researchers about the goodness of fit, Bagozzi and Yi (1988) indicate that the standardized factor loading value should not be under 0.6 or higher than 0.95, and that the value of the construct reliability (CR) should be higher than 0.7. Further, the value of average variance extracted (AVE) should be higher than 0.5, and the loading value for each measurement item should be above 0.6. If these standards are achieved, the item should be retained and the collected data confirmed as valid. Where standard values are not achieved, the item should be removed from the analysis. The results of this study are shown in Supplementary Table 2, which indicate that the convergent validity is good.

#### Discriminant validity analysis

The discriminant validity analysis is employed primarily to ensure that the latent variables used for measuring the causal relationships under study are truly different from each other (Fornell and Larcker, 1981). To assess the discriminant validity of the concepts, the square root of AVE must be significantly larger than the correlation between the construct and other constructs. If the square of the correlation coefficient is not greater than the AVE, it can be considered that there is good discriminant validity. The results are outlined in Supplementary Table 3, with the diagonal values being the AVE values for every dimension. Thus, the discriminant validity of each concept is judged to be good.

### Results of hypotheses test

#### Results of path analysis

For hypothesis testing, a simple regression analysis was performed and the results shown in Supplementary Table 4. Our test results all support H1, H2, and H3. With regard to H1, the analysis results show that the explanatory power of the regression model is 33.5%, and dysfunctional consumer behavior (DCB) was shown to have a statistically significant positive (+) effect on an employee’s negative emotions (β = 0.579, *p* < 0.001). This means that when DCB increased, the employee’s negative emotions increased, and, thus, Hypothesis1 is accepted. This result is consistent with Han et al.’s (2016) work, that investigated the role of customer misbehavior in the setting of a national retail store, the results of which illustrated that the frequency of customer misbehavior significantly increases the levels of employee negative emotions.

Results also indicate that dysfunctional customer behavior is significantly and negatively associated with employee prosocial service behavior (β = −0.287, *p* < 0.001), and that negative emotions triggered by dysfunctional customer behaviors have a negative impact on employees’ prosocial service behavior (β = −0.375, *p* < 0.001). Therefore, H2 and H3 are accepted.

#### Results of the mediating effect of negative emotion

Hypothesis 4 anticipated that negative emotion plays a significant mediating role in the relationship between dysfunctional customer behaviors and frontline employees’ prosocial service behavior (PSB). The mediating effect of negative emotions is verified through SPSS Process macro model 4.

Firstly, the verification results of the mediation effect of negative emotion (NE) in the DCB and PSB relationship are shown in Supplementary Table 5. DCB has been shown to have a significantly negative effect on PSB (β = −0.287, *p* < 0.001). This means that, if frontline employees experience considerable dysfunctional customer behavior, their intention to conduct prosocial service behaviors will reduce. The explanatory power of the PSB for DCB is 8.2% (R^2^ = 0.082). Next, DCB has been shown to have a significantly positive effect on NE (negative emotion; β = 0.579, *p* < 0.001), which means that, if frontline employees experience considerable dysfunctional customer behavior, they would experience more negative emotions, with an explanatory power of 33.5% (R^2^ = 0.335). Finally, when the effect of NE on the PSB with the DCB is under control (β = −0.314, *p* < 0.001), it was found that the higher the NE, the lower the PSB. In considering the effect of DCB on the PSB while controlling the NE, we confirm that the effect of DCB on the PSB is not significant (β = −0.105, *p* > 0.05), which means that NE has a full mediation effect in the relationship between DCB and PSB. The explanatory power of the PSB on DCB and NE is 14.8% (R^2^ = 0.148).

Next, bootstrapping was performed to confirm the significance of the indirect effect. The results are shown in Supplementary Table 6. When NE was mediated in the relationship between DCB and PSB, a confidence interval of 95% for the indirect effect of the path DCB → NE → PSB was −0.184 to −0.052. The indirect effect was significant because this range did not include zero. This means that NE mediates in the relationship between DCB and PSB. The direct effect of the path DCB → PSB was not significant, and, at a confidence interval of 95%, was found to be −0.172 to −0.039 and did not contain zero. Thus, the results confirm that NE is fully mediated in the relationship between DCB and PSB. These findings further confirm that DCB does not have a direct effect on PSB, but the more that frontline employees experience DCB, the higher the NE they will feel, thereby further reducing employees’ intention for PSB.

#### Results of the moderating effect of perceived supervisor support

To confirm the moderating effect of perceived supervisor support (PSS), a hierarchical regression analysis was performed. To reduce any multicollinearity problems that might arise, the mean centering of the variables was calculated to analyze the effect of the interaction term of the independent and moderator variables. In step 1, the independent variable DCB and the moderator variable PSS are added to verify the significance effect on the dependent variable “negative emotion.” In step 2, the interaction terms of the independent variable DCB and the moderator variable PSS are added to verify the effect on negative emotion, the dependent variable. The amount of change in R^2^ was statistically significant (△*R*^2^ = 0.015, *p* < 0.05). This means that the relationship between dysfunctional customer behavior and negative emotion depends on the level of PSS. That is, as the perceived level of superior support changes, the relationship between DCB and NE also changed. The results are presented in Supplementary Table 7.

As Aiken et al. (1991) suggest, the significance of the interaction was verified using the average value of perceived supervisor support and the ±1SD value. The results are presented in Supplementary Table 8. A simple regression line showing the relationship of DCB to negative emotion showed significant results in both the mean value of PSS and the value of ±1SD. This means that the relationship between dysfunctional customer behavior and negative emotion is significantly moderated by perceived supervisor support.

## Discussion

### Conclusion and application

A characteristic of the service industry is the requirement of frontline employees to perform emotional labor when they serve customers, and from which they frequently experience emotional dissonance and emotional exhaustion. In the event that employees deal with the misbehavior of customers, the negative emotions they experience, such as fatigue, frustration, and anger can cause long-term psychological stress and mental pressure. These negative emotions can have a negative impact on the employee’s subsequent service behavior. This study conducted a survey of bank staff as the research object to verify the mediating effect of negative emotions in the relationship between dysfunctional customer behavior and employees’ prosocial service behavior. It also sought to test whether perceived supervisor support plays an effective role in regulating employee negative emotions when triggered by customer misbehavior. The main findings of the study are discussed here.

Past research strongly suggests that frontline service employees’ negative emotions are an important consequence of dysfunctional customer behavior (Harris and Reynolds, 2004; Harris and Daunt, 2013). In our current study, dysfunctional customer behavior has been shown to increase negative emotions and decrease the prosocial service behavior intentions of frontline bank service employees. The effect of dysfunctional customer behavior on negative emotions was found to be significantly positive. This is consistent with the findings of Huang and Miao (2016) indicating that dysfunctional customer behavior has a powerful effect on the negative emotions of frontline employees. In other words, the more dysfunctional customer behavior service employees perceive or experience, the more negative emotions they feel. Moreover, results show that dysfunctional customer behavior had a significant negative impact on the prosocial service behavior of bank service employees. Further, the analysis of the effect of negative emotions on prosocial service behavior intentions shows that the negative emotions of employees decrease their intention to provide customers with prosocial service, meaning that the higher the employee perception of negative emotions, the less they desire to provide customer-oriented service or to help other coworkers. To motivate employees to conduct prosocial service behavior, it is necessary to manage their negative emotions.

The mediating effect that negative emotions played in the relationship between dysfunctional customer behavior and prosocial service behavior was verified and found to be fully mediated. The results confirmed that dysfunctional customer behavior affected an employee’s intention to provide customer prosocial service *via* their negative emotional state. Shao and Skarlicki (2014) found that employees who encountered dysfunctional customer behavior lost resources, leading them to engage in sabotage action against customers to compensate for their own loss, i.e., they were unwilling to exhibit prosocial behavior. Our study begins from the perspective of prosocial service behavior, and the empirical results show that employees’ prosocial service behavior can be suppressed through the influence of negative emotion. This finding provides insight into the reason for employees to reject prosocial behaviors as stemming from customer misbehavior and establishes the need to identify and prevent customer misbehavior to avoid its harmful effects. The study results show that perceived supervisor support has a moderating effect on the relationship between dysfunctional customer behavior and negative emotion.

These findings suggest that dysfunctional customer behavior affects the emotions and behaviors of employees, which illustrates the importance of managing dysfunctional customer behavior. For managers, understanding the negative impact of dysfunctional customer behavior and actively managing these behaviors will improve enterprise performance. For instance, to prevent customer dysfunctional behavior, managing customer behavior would include posting notices in the workplace, improving customer information systems, formulating manuals for responding to different types of dysfunctional customer behavior, and conducting training for employees on how to deal with customer misbehavior. Thus, when such a situation occurs, a strong response should be taken in accordance with the guidelines to prevent recurrence.

With regard to the impact of negative emotions, managers need to pay more attention to the negative emotions of employees, and firms should establish effective internal marketing strategies to manage employee emotions. Negative emotion management can be divided into, firstly, preventing negative emotions from occurring and, secondly, strategies to solve problems when negative emotions occur. Such management measures include, for example, communicating with employees, understanding the factors that cause employee negative emotions, and improving these factors to prevent or reduce the occurrence of negative emotions. In addition, firms should first break the curse of “the customer is king” and “customers are always right,” understanding instead that blindly allowing employees to unconditionally tolerate customers’ poor behaviors has a negative impact on firm performance. Through training to impart appropriate emotional regulation methods and establish appropriate reward mechanisms, the negative emotions of employees can be alleviated. To heal emotions after experiencing negative emotions, positive support activities, such as treatment and counseling support, healing programs, and leisure activities can help employees alleviate or eliminate negative emotions when they arise.

As perceived supervisor support can reduce employee negative emotions triggered by dysfunctional customer behavior, managers should recognize and acknowledge the importance of supervisor support. When employees feel a high level of supervisor support, the impact of dysfunctional customer behavior on employees’ negative emotions is weakened, and, conversely, when employees perceive a sense of low supervisor support, the impact of customer misbehavior on employees’ negative emotions is enhanced. From a practical point of view, managers should take positive and effective measures to enhance perceived supervisor support of employees to help them recover from negative emotions when they arise. For example, when customer misbehavior in the workplace occurs, the manager should promptly assist the field service employee to solve the problem and give timely and necessary task support; and when customer misbehavior causes physical and psychological harm to the employee, supervisors should provide timely comfort and encouragement, and material and spiritual support. Moreover, understanding and supporting the employee experiencing difficulties at work or in life will allow the employee to feel the care and warmth of their supervisor; by carefully listening to and considering the ideas and opinions put forward by employees, the employee’s sense of senior management support can be improved. This will help employees to eliminate and recover from negative emotions quickly.

This study will contribute to both theory and practice. From a theoretical point of view, it is conducive, firstly, to deepening the understanding of customer misconduct. The study divides dysfunctional customer behavior into three categories: insulting words, rude behavior, and rude attitudes, thereby expanding the breadth of understanding of what customer misconduct entails based on empirical evidence. Thus, it provides new ideas and a research foundation for comprehensively uncovering the emotional impact of dysfunctional customer behavior on employees. Secondly, the study explores the ways in which dysfunctional customer behavior affects the service behavior of frontline service staff. Through empirical investigation, the study will trace the transmission path and influence relationship between dysfunctional customer behavior, negative emotions, and prosocial service behavior, to provide a theoretical foundation for subsequent research and follow-up studies allowing a more accurate formulation of the relationship between these three factors. Thirdly, the study clarifies how negative emotions have an intermediary effect on the relationship between dysfunctional customer behavior and prosocial service behavior and investigates the predictive effect of negative emotions on prosocial service behavior. Negative emotions can compensate for an employee’s inability to express dissatisfaction and distress brought about by the externally derived dysfunctional behavior of customers, which, in turn, can affect employees’ follow-up service behavior, a topic absent from the current literature on employee service effectiveness. Fourthly, the study reveals the regulatory role of perceived supervisor support in moderating the negative emotional impact of customer misbehavior on front-line employees and assesses the ways in which perceived supervisor support can alleviate the negative emotions of service employees when encountering dysfunctional customer behavior. These research results will broaden the research horizon relating to employee coping strategies and adjustment mechanisms in response to customer misbehavior.

In terms of practical significance, the study results will serve as a reminder to service enterprises to pay attention to the emotional health of front-line employees, in particular to provide training to ensure appropriateness-of-fit when recruiting and assessing new employees’ suitability for front-line work. It is common for front-line employees of service enterprises to experience dysfunctional customer behavior, which will inevitably affect their emotions. This may, in turn, affect customers’ perceptions of service quality and thus customer satisfaction, that may have an impact on the competitiveness of service enterprises. From the perspective of emotion, based on empirical analysis, this study discusses the mechanism through which dysfunctional customer behavior impacts on the (negative) emotions of front-line service employees. It proposes mitigation strategies and recommendations which reveal the formation process of the effect of dysfunctional customer behavior as negative emotions in front-line service employees, clarifies the reasons for employees’ negative emotions, and enables enterprise managers to deepen their understanding of front-line employee responses to customer misconduct. Critically, the study will elucidate why, should service firms continually ignore the existence of dysfunctional customer behavior, lose their voice in the face of customer misconduct, or fail to provide front-line service employees with appropriate supervisor support and understanding, enterprise shortcomings will lead to negative emotions of front-line service employees.

The study calls on service firms to change their marketing orientation from “the customers are always right” to “treat customers correctly” to encourage service firms to attach importance to the emotional health of front-line service employees, enhance their sense of belonging, and instill happiness in front-line employees from their work by taking appropriate control measures to respond to dysfunctional customer behavior. At the same time, service-oriented enterprises need to undertake psychological interventions and training for front-line service employees to improve their skills in identifying customer misconduct and their flexibility in dealing with emergencies, to reduce the possibility of negative impact due to negative employee emotions. The results will induce accurate recruitment, assessment, and training of front-line service employees by service-oriented firms, thereby laying the foundation for selecting employees suitable for the work conditions and requirements of front-line positions and improving the overall service quality of enterprises. It will further induce front-line service employees to improve their ability to deal with dysfunctional customer behavior and to manage their own stress and negative emotions.

By discussing the manifestation of dysfunctional customer behavior in banking services and its impact on frontline service employees in the form the negative emotions, this paper will assist frontline employees to recognize types of dysfunctional customer behavior, master strategies to deal with customer misconduct, and improve their ability to effectively face and resolve the pressure from, and negative emotional impact of, customer misconduct. In this regard, the study finds that bank managers should pay conscious attention to contact between employees and customers in service areas and provide the necessary support to subordinates in a more timely and effective manner.

Finally, the study identifies opportunities for customer empathy and avenues to reduce the occurrence of misbehavior. As a customer who is in direct contact with front-line employees of service-oriented enterprises, it is likely that the customers themselves, their families, or their friends are also engaged as front-line employees of service-oriented enterprises. This study may have a “knock-on” effect in prompting customers to reflect on the appropriateness of their behavior in the process of receiving services, thereby reducing the likelihood of dysfunctional customer behavior, its emotional toll on employees, and its economic cost on service enterprises.

### Limitations and recommendations

There were limitations to the study. Firstly, the sample was limited. The research sample for this study is restricted to Qingdao City in China and the representativeness of the sample for overall bank service employees is unknown. Moreover, in different service scenarios, the degree of interaction between frontline service employees and customers differs, and the results of future research may also differ from those of this study. Therefore, the study results may not be applicable within a general service context. Because our sample of respondents are Chinese, our findings may not be generalizable to other cultural contexts. Future research could explore the mechanisms of dysfunctional customer behavior, negative emotion, and prosocial service behavior within a cross-cultural context. Further, the data used for statistical analysis was collected over a relatively short period of time and the sample size was relatively small. Although sufficient and satisfactory answers were obtained using statistical estimates with a 95% confidence level and with ±0.05 error in statistical analysis, for future studies, a larger sample is recommended to conduct empirical studies with statistical confidence.

Dysfunctional customer behavior is widespread and diverse within service interactions according to the classification of problematic customers identified in earlier marketing research. This includes the aggressive behavior that has emerged in recent years as indicated by the results of organizational behavior research, all of which reflect the complexity and diversity of the real world. Another limitation of the study is the dysfunctional customer behavior scales adopted and used, which were developed from a service context perspective, and these behaviors may not be an accurate reflection of the bank-specific environment. Therefore, we suggest that future research should develop a scale for bank-specific customer misbehavior to provide more targeted recommendations for bank managers; for example, how to predict and prevent customer dysfunctional behaviors in advance, and how bank employees can deal with these dysfunctional customer behaviors. Furthermore, on combing through the research results of relevant literature this research found that, in addition to the three behaviors of insulting words, rude behavior, and rude attitudes covered in this article, there were also behaviors such as customer threats, assault, and other related behaviors. In addition, with the rapid development of online shopping, some behaviors commonly violate social norms, such as posting deliberate negative comments or reviews. However, research in this area is rare and there is as yet no well-developed scales to apply. Hence, this paper has not included all forms of customer misconduct in these new business forms within the research category. This can be taken as the direction of future research.

Finally, this study identified customer misbehavior during the service process as the main cause of negative emotions among employees. In fact, for frontline service employees, the causes of their negative emotions are various, and it is necessary for future research to explore other variables causing negative emotions among employees. In addition, this study proposed that perceived supervisor support can effectively regulate employees’ negative emotions triggered by customer misbehavior, and future research should explore strategies and methods for preventing and alleviating employees’ negative emotions. In other words, future research can integrate mediating variables and regulatory variables from a variety of theoretical perspectives to more deeply reveal how customer misbehaviors affect employee psychology, their attitudes and behaviors, which can make the research model more rigorous. In conclusion, negative emotion is a subjective evaluation, which is inevitably affected by individual factors that cannot be objectively revealed. In future, psychological research methods will describe the stress state of the subject by observing the physiological response of the subject.

## Data availability statement

The original contributions presented in the study are included in the article/Supplementary material, further inquiries can be directed to the corresponding author.

## Ethics statement

Ethical review and approval was not required for the study on human participants in accordance with the local legislation and institutional requirements. Written informed consent from the patients/participants or patients/participants legal guardian/next of kin was not required to participate in this study in accordance with the national legislation and the institutional requirements.

## Author contributions

BX designed the research and wrote the manuscript. CL collected literature. XZ and BX performed the empirical analysis. YL provided the data and polished the article. All authors contributed to the article and approved the submitted version.

## Funding

This research was funded by the Natural Science Foundation of Shandong Province (ZR2020MG004).

## Conflict of interest

The authors declare that the research was conducted in the absence of any commercial or financial relationships that could be construed as a potential conflict of interest.

## Publisher’s note

All claims expressed in this article are solely those of the authors and do not necessarily represent those of their affiliated organizations, or those of the publisher, the editors and the reviewers. Any product that may be evaluated in this article, or claim that may be made by its manufacturer, is not guaranteed or endorsed by the publisher.
