# Supplementary material for: Service staff encounters with dysfunctional customer behavior: Does supervisor support mitigate negative emotions?
Source: Front Psychol. 2022 Aug 29;13:987428. doi: 10.3389/fpsyg.2022.987428 (PMC9465484; doi:10.3389/fpsyg.2022.987428)
Supplement: Supplementary file 1 [file Data_Sheet_1.docx]

Supplementary Material

# Supplementary Figures and Tables

## Supplementary Figures

Dysfunctional

Customer Behavior

Prosocial

Service Behavior

Negative Emotion

Perceived

Supervisor

Support

Figure 1. Research framework

## Supplementary Tables

Table 1 The results of reliability analysis

| **Variable** | | **Mean** | **SD** | **Cronbach’s α** |
| --- | --- | --- | --- | --- |
| Dysfunctional customer behavior | | 2.643 | 0.718 | 0.903 |
| Negative emotion | | 2.365 | 0.760 | 0.959 |
| Prosocial service behavior | PSB1 | 4.281 | 0.493 | 0.874 |
|  | PSB2 | 4.199 | 0.581 | 0.907 |
|  | PSB3 | 3.316 | 0.719 | 0.855 |
| Perceived supervisor support | | 2.245 | 0.765 | 0.887 |

Table2 The results of convergent validity analysis

| **Concept** | | **Measuring items** | **Loading Value** | **Cronbach's α** | **C.R** | **AVE** |
| --- | --- | --- | --- | --- | --- | --- |
| Dysfunctional customer behavior | | DCB3 | 0.739 | 0.903 | 0.923 | 0.633 |
|  |  | DCB4 | 0.648 |  |  |  |
|  |  | DCB5 | 0.815 |  |  |  |
|  |  | DCB6 | 0.845 |  |  |  |
|  |  | DCB7 | 0.793 |  |  |  |
|  |  | DCB8 | 0.830 |  |  |  |
|  |  | DCB9 | 0.620 |  |  |  |
| Negative emotion | | NE1 | 0.660 | 0.959 | 0.963 | 0.635 |
|  |  | NE2 | 0.666 |  |  |  |
|  |  | NE3 | 0.745 |  |  |  |
|  |  | NE4 | 0.671 |  |  |  |
|  |  | NE5 | 0.791 |  |  |  |
|  |  | NE6 | 0.768 |  |  |  |
|  |  | NE7 | 0.758 |  |  |  |
|  |  | NE8 | 0.850 |  |  |  |
|  |  | NE9 | 0.840 |  |  |  |
|  |  | NE10 | 0.882 |  |  |  |
|  |  | NE11 | 0.819 |  |  |  |
|  |  | NE12 | 0.903 |  |  |  |
|  |  | NE13 | 0.634 |  |  |  |
|  |  | NE14 | 0.852 |  |  |  |
|  |  | NE15 | 0.837 |  |  |  |
| Perceived supervisor support | | PSS1 | 0.632 | 0.887 | 0.912 | 0.725 |
|  |  | PSS2 | 0.849 |  |  |  |
|  |  | PSS3 | 0.916 |  |  |  |
|  |  | PSS4 | 0.883 |  |  |  |
| PSB | PSB1 | PSB11 | 0.756 | 0.874 | 0.952 | 0.799 |
|  |  | PSB12 | 0.810 |  |  |  |
|  |  | PSB13 | 0.846 |  |  |  |
|  |  | PSB14 | 0.735 |  |  |  |
|  |  | PSB15 | 0.678 |  |  |  |
|  | PSB2 | PSB21 | 0.780 | 0.907 | 0.956 | 0.814 |
|  |  | PSB22 | 0.801 |  |  |  |
|  |  | PSB23 | 0.861 |  |  |  |
|  |  | PSB24 | 0.854 |  |  |  |
|  |  | PSB25 | 0.792 |  |  |  |
|  | PSB3 | PSB32 | 0.684 | 0.855 | 0.893 | 0.679 |
|  |  | PSB33 | 0.813 |  |  |  |
|  |  | PSB34 | 0.907 |  |  |  |
|  |  | PSB35 | 0.697 |  |  |  |

Note: DCB: dysfunctional customer behavior; PSB: prosocial service behavior; NE: negative emotion; PSS: perceived supervisor support.

Table3 Discriminant validity

| Concepts | | DCB | NE | PSS | PSB | | |
| --- | --- | --- | --- | --- | --- | --- | --- |
|  |  |  |  |  | PSB1 | PSB2 | PSB3 |
| DCB | | 0.633 |  |  |  |  |  |
| NE | | .579** | 0.635 |  |  |  |  |
| PSS | | 0.112 | -0.013 | 0.725 |  |  |  |
| PSB | PSB1 | -.252** | -0.368 ** | 0.051 | 0.799 |  |  |
|  | PSB2 | -.183* | -0.357** | 0.034 | 0.629 ** | 0.814 |  |
|  | PSB3 | -.226** | -0.174** | -0.076 | 0.218** | 0.22** | 0.679 |

Note: ***p<.001, **p<.01; DCB: dysfunctional customer behavior; PSB: prosocial service behavior; NE: negative emotion; PSS: perceived supervisor support.

Table 4 The results of structural equation modeling path analysis

| **Hypothesis** | **Path** | **B** | **SE** | **β** | **t** | **P** | **F** | **R^2^** | **Result** |
| --- | --- | --- | --- | --- | --- | --- | --- | --- | --- |
| H1 | constant | 0.745 | 0.175 |  | 4.266 | 0.000 | 92.214 | 0.335 | Accepted |
|  | DCB→NE | 0.613 | 0.064 | 0.579 | 9.603 | 0.000 |  |  |  |
| H2 | constant | 4.414 | 0.123 |  | 35.755 | 0.000 | 16.373 | 0.082 | Accepted |
|  | DCB→PSB | -0.182 | 0.045 | -0.287 | -4.046 | 0.000 |  |  |  |
| H3 | constant | 4.465 | 0.102 |  | 43.624 | .0000 | 29.909 | 0.140 | Accepted |
|  | NE→PSB | -0.225 | 0.041 | -0.375 | -5.469 | 0.000 |  |  |  |

Note: DCB: dysfunctional customer behavior; PSB: prosocial service behavior; NE: negative emotion.

Table 5 Results of the mediating effect of negative emotion

| **Independent**  **variable** | **Dependent**  **variable** | **B** | **SE** | **β** | **t** | **R^2^** | **F** |
| --- | --- | --- | --- | --- | --- | --- | --- |
|  |  |  |  |  |  |  |  |
| DCB | PSB | -0.183 | 0.045 | -0.287 | -4.064*** | 0.082 | 16.373 |
| DCB | NE | 0.613 | 0.064 | 0.579 | 9.603*** | 0.335 | 92.213 |
| DCB | PSB | -0.067 | 0.053 | -0.105 | -1.247 | 0.148 | 16.8159 |
| NE |  | -0.189 | 0.051 | -0.314 | -3.744*** |  |  |

Note: ***p<.001; DCB: dysfunctional customer behavior; PSB: prosocial service behavior; NE: negative emotion.

Table 6 Bootstrapping results for the indirect effect of negative emotions

| Path | Effect(p) | SE | 95% confidence interval | |
| --- | --- | --- | --- | --- |
|  |  |  | LLCI | ULCI |
| Total effect  DCB→PSB | -0.183(0.001) | 0.045 | -0.271 | -0.094 |
| Direct effect  DCB→PSB | -0.067(0.214) | 0.053 | -0.172 | 0.039 |
| Indirect effect  DCB→NE→PSB | -0.116 | 0.034 | -0.184 | -0.052 |

Note: ***p<.001; LLCL: Lower limit within the 95% confidence interval; ULCL: Upper limit within the 95% confidence interval; DCB: dysfunctional customer behavior; PSB: prosocial service behavior; NE: negative emotion.

Table 7 The moderating effect of perceived supervisor support

| Dependent variable: negative emotion | | | | | | | | |
| --- | --- | --- | --- | --- | --- | --- | --- | --- |
| Step | variable | Unstandardized coefficient | | β | t | R^2^ | △R^2^ |  |
|  |  | B | SE |  |  |  |  |  |
| 1 | Constant | 0.896 | 0.210 |  | 4.274 | 0.341*** |  |  |
|  | DCB | 0.622 | 0.064 | 0.588 | 9.705 |  |  |  |
|  | PSS | -0.078 | 0.060 | -0.078 | -1.295 |  |  |  |
| 2 | Constant | 2.013 | 0.575 |  | 3.501 | 0.357*** | 0.015* |  |
|  | DCB | 0.191 | 0.216 | 0.180 | 0.882 |  |  |  |
|  | PSS | -0.587 | 0.251 | -0.590 | -2.334 |  |  |  |
|  | DCB×PSS | 0.194 | 0.093 | 0.700 | 2.083 |  |  |  |

Note: ***p<.001, **p<.01, *p<.05; DCB: dysfunctional customer behavior, PSS: perceived supervisor support

Table 8 Verification of Significance for the conditional effects of PSS

|  |  | Effect | SE | t | LLCL | ULCL |
| --- | --- | --- | --- | --- | --- | --- |
| Perceived supervisor support(PSS) | -ISD | 0.479 | 0.094 | 5.109*** | 0.294 | 0.664 |
|  | M | 0.627 | 0.064 | 9.868*** | 0.502 | 0.753 |
|  | +ISD | 0.776 | 0.097 | 7.966*** | 0.584 | 0.968 |

Note: ***p<.001, LLCL: Lower limit within the 95% confidence interval; ULCL: Upper limit within the 95% confidence interval.
